# Managing Retinitis Pigmentosa: A Literature Review of Current Non-Surgical Approaches

**DOI:** 10.3390/jcm14020330

**Published:** 2025-01-08

**Authors:** Leonardo Colombo, Jacopo Baldesi, Salvatore Martella, Chiara Quisisana, Aleksei Antico, Luca Mapelli, Stefania Montagner, Alberto Primon, Luca Rossetti

**Affiliations:** 1 Eye Clinic, ASST Santi Paolo e Carlo Hospital, University of Milan, 20142 Milan, Italy; jacopo.baldesi@unimi.it (J.B.); salvatore.martella@unimi.it (S.M.); chiara.quisisana@unimi.it (C.Q.); alekseiantico110@gmail.com (A.A.); lmapelli1424@gmail.com (L.M.); luca.rossetti@unimi.it (L.R.); 2 Eye & Vision—Visual Rehabilitation Center, 20128 Milan, Italy; stefania.montagner@eyevision.it (S.M.); alberto.primon@eyevision.it (A.P.)

**Keywords:** retinitis pigmentosa, multidisciplinary approach, visual rehabilitation, non-surgical interventions

## Abstract

Retinitis pigmentosa (RP) is a heterogeneous group of inherited retinal diseases characterized by the progressive loss of photoreceptor function, visual impairment, and, ultimately, blindness. While gene therapy has emerged as a promising therapy, it is currently available only for the RPE65 gene mutation, leaving many patients without targeted genetic treatments. Non-surgical interventions may help in managing the progression of RP and improving patients’ quality of life. Visual training and rehabilitation, maximizing residual vision, have shown potential in improving mobility and patients’ ability to perform daily activities. Visual aids enhance visual function. Moreover, photo-protection demonstrated effectiveness in mitigating light-induced damage and improving visual comfort. Alternative therapies (i.e., electrostimulation, acupuncture, and ozone therapy) are being explored to preserve retinal function and reduce disease progression. Pharmacological interventions supported by nutritional and psychological counseling play a role in slowing retinal degeneration while managing the emotional burden of progressive vision loss. Although for these interventions, further validation is required, their potential benefits make them valuable additions to care for RP patients. The integration of these interventions into a multidisciplinary care approach—including ophthalmologists, orthoptist, dietitians, and psychologists—is essential for providing comprehensive, personalized care to RP patients while awaiting more widespread gene therapy solutions.

## 1. Introduction

Retinitis pigmentosa (RP) is a genetic eye disorder belonging to a heterogeneous group of inherited retinal diseases [1]. Genetic variants in over 80 genes with more than 3100 mutations are recognized as drivers of RP, which is characterized by the gradual deterioration of the retina’s photoreceptor cells [1,2]. The symptoms typically begin in childhood or adolescence and result in visual impairment including night blindness, peripheral limitations and/or scotomas in the visual field (VF), frequent reduction in visual acuity (VA), and alterations of contrast sensitivity (CS) [1,3,4,5,6]. RP affects approximately 1 in 5000 people worldwide [7]. Different biochemical pathways of rod photoreceptors may be impaired in RP individuals due to the different genetic mutations responsible for the onset of the disease [1]. Apoptosis, necrosis, and autophagy cause increased disruptions of the retinal pigment epithelium, which lead to the gradual progression of the disease [1].

Disease progression is associated with a significant impact on the patients’ quality of life (QoL) [8]. RP visually impaired patients likely experience a reduced degree of independence in their personal life and can face serious difficulties in daily activities, with consequences on lifestyle, loss of productive hours, social development, well-being, and psychopathological outcome [8]. Maintaining the ability to manage personal life is crucial for patients’ QoL.

Ongoing research is dedicated to the development of innovative approaches to manage inherited retinal diseases, such as advanced genetic therapy. Indeed, for patients affected by RPE65-related retinal dystrophy, gene therapy is currently available [9]. No specific gene therapy is yet available for the treatment of other forms of RP, but several pharmacological and non-pharmacological interventions could be combined to reach an effective management method of the disease and to increase patients’ QoL, according to the degree of vision loss and the patient’s needs and characteristics.

Here, we propose a critical review of the current available evidence about non-surgical treatments that can be applied in the clinical management of RP patients. For the purposes of this review, surgical therapeutic approaches (including gene therapy, optogenetics, artificial retinal implants, etc.) and the treatment of RP complications, such as macular edema, epiretinal membranes, or early cataracts, will not be considered.

## 2. Enhancing Vision in Retinitis Pigmentosa

### 2.1. Visual Training

Given the gradual progression of RP, early interventions may be the most effective. Visual training and rehabilitation can support individuals to maximize their remaining vision thus improving their ability to deal with daily tasks. Extensive research has focused on understanding the visual search, gaze behavior, ability of RP patients to perform environmental analysis, and the mental effort required for walking in order to identify techniques aimed at enhancing their functionality despite vision loss.

Through the use of a video camera simultaneously recording patient’s eye movements and the observed environment, Turano and coworkers highlighted how the visual impairment influenced the ability to analyze the surrounding environment [10]. Moreover, RP patients displayed a longer reaction time and more mental effort when moving through a complex route [11]. Geruschat et al. [12] showed that patients with RP move slower than healthy individuals. The longer time needed to react to a stimulus and to navigate the space may be related to the effort required to analyze the surroundings due to visual impairment. Although RP patients were shown to have more active visual search patterns [13], increased scanning eye movements do not compensate for the missing information due to peripheral field loss [14].

Interventions that can support RP patients to better orient themselves in their surroundings to enhance mobility and safety have been proposed. For example, Peli et al. [15] analyzed the binocular residual peripheral islands of patients with RP and used the mathematical modeling of the VF expansion to model the reduction in the collision risk density. Indeed, field expansion devices, particularly prisms that create artificial peripheral islands at optimal angles, can enhance hazard detection and reduce pedestrian collision risks for individuals with peripheral visual field loss [15]. Eye movement training can improve the mobility. In a pilot study, the oculomotor responses during walking and obstacle avoidance in a controlled environment were evaluated both before and after specific training. The training was beneficial for RP patients and resulted in shorter fixation durations; also, a significant improvement in relative walking speed during navigation in a real-world like controlled environment was noted in this study [16].

A visual training protocol, based on software generating light stimuli of different durations and intensities, recording the stimuli perceived by the subject was applied to RP patients with reduced contrast sensitivity [17]. The results showed that the experimental group made significant progress in all variables, which remained stable after 3 months, suggesting that the program could be a useful rehabilitation for RP patients and help mitigate the damage.

All these supports are still under development and are not yet widely used in clinical practice. Further research and clinical trials are needed to validate their effectiveness and to develop specific validated protocols to be integrated into standard care for RP patients.

### 2.2. Visual Aids: From Traditional Magnifiers to Advanced Digital Solutions

One of the primary objectives in managing patients with extremely low vision is to provide them with appropriate aids. Magnifying glasses are useful aids for patients with high visual impairments. The increase in the spherical power provided by these aids comes with a reduction in the VF, an important limitation to patients suffering from RP. High-magnification reading aids may be less effective for patients with severe VF constriction [18].

New technologies, such as digital tools, devices, and software, have been explored to provide support in maintaining some degree of independence, enabling these patients to perform daily activities. One example is the video magnifier, a device that captures live images with a camera, magnifies them, and displays them on a screen, making it easier for the user to see. The reversed polarity visualization provided by this tool adjusts the color of the image to enhance contrast. This high-contrast mode enhances readability for people with visual impairments by reducing glare and improving legibility. The integration of a speech synthesis program is particularly useful in patients with total blindness, enabling them to access written content through auditory means [19].

Aids for RP patients are not only limited to video magnifiers and speech synthesis, but also to orientation and mobility devices: in a pilot study conducted on twenty-six subjects, twenty healthy and six with RP, a Low-vision Enhancement Optoelectronic (LEO) belt was able to increase mobility accuracy and self-confidence when navigating a maze. However, regardless of the severity of vision loss or simulated vision loss, all subjects were slower to complete mazes using the device.

The positive feedback from users suggests that the LEO belt has the potential to become another visual aid for visually impaired individuals, especially those with visual field damage, but further studies on a larger number of patients are needed [20].

Advances in technology provide visually impaired patients with an ever-increasing range of possibilities to improve the independence of many daily-life activities. Furthermore, the advent of artificial intelligence will open up scenarios yet to be explored; in light of these considerations, the presence of a low vision center for the study and customized application of visual aids plays a fundamental role in the management of RP patients.

### 2.3. Photoprotection

Photoprotection refers to the use of specific filters or lenses to reduce the harmful effects of light on the retina. Photoprotection is particularly important for RP patients, as excessive exposure to certain wavelengths of light can accelerate the progression of the disease. As blue-violet light is the interval of visible spectrum associated with the highest energy content, it has historically been considered playing a major role in causing photophobia, glare vision, and inducing intraocular scattering [21,22], influencing vision and the QoL of patients affected by retinal dystrophies.

Several studies have underlined how the use of a specific filter in RP patients can improve their discomfort with light [23,24,25]. Functional visual parameters have been assessed using photochromic and selective blue–violet light filtering spectacle lenses in patients experiencing central or peripheral scotoma, as blue light was associated with photochemical damage to retinal tissue and retinal pigment epithelium (RPE). Blue-violet light filtering lenses enhanced BCVA, CS, and glare in all evaluated patients [23]. More specifically, some studies showed that, while longer blue wavelengths of the visible spectrum (465–495 nm) are essential to regulate circadian rhythm and to ensure a normal visual function [26,27], blue-violet light (380–465 nm) could cause an oxygen-dependent retinal injury acting on specific chromophores (by-products of visual cycle) [28,29].

Otsuka Y. et al. [24] aimed to identify the primary wavelength of light responsible for photophobia in inherited retinal diseases. Patients with RP showed better relief with short wavelength filtering glasses, while in cone–rod dystrophy (CRD) patients, no significant difference could be recorded with either short- or middle-wavelength glasses [24]. The findings suggest that photophobia in RP patients is primarily induced by short-wavelength light, whereas both short- and middle-wavelength light contributed to photophobia in patients with CRD. Different wavelengths of light caused photophobia depending on the disease and probably on the pathological condition and disease stage. Indeed, Cedrùn-Sànchez et al. [25] evaluated RP patients under low illumination conditions, demonstrating that short-wavelength light absorbance filters increased visual discrimination ability [25].

The use of adequate filters in patients with specific retinal pathologies can thus increase visual functionality and improve visual discomfort. The evaluation and prescription of filters should be considered in the clinical practice.

## 3. Complementary Therapies

### 3.1. Electrical Stimulation

Electrical stimulation (ES) is a non-pharmacological technique based on microcurrent delivery to a target tissue. Microcurrent induces biochemical effects on the cells that may help preserve or restore vision [30,31]. The first reported application of ES to the eyes dates back to 1755, when Charles LeRoy applied an electrical discharge to the eye of a cataract-induced blind patient, resulting in visual sensations [32]. In 2004, Chow et al. showed retinal visual improvement in RP patients carrying a sub-retinal prosthesis which produced sub-threshold currents, even in the area of the retina far from the implant [33]. They hypothesized that the chronic low-level electrical stimulation of a partially degenerated retina induces the release of neurotrophic factors which enhance the function of the remaining, inadequately functioning photoreceptors [33]. From then on, numerous studies have been conducted to explore the therapeutic potential of ES on a damaged retina, focusing on the biochemical changes induced by ES. Preclinical studies in animal models of photoreceptor degenerations helped to identify the biochemical pathways involved by ES stimulation: the inhibition of neuronal apoptosis, an increase in the ciliary neurotrophic factor and brain-derived neurotrophic factor (BDNF), the induction of neuronal degeneration, and the modulation of brain plasticity [34,35,36,37].

To date, no clinical studies have been conducted on RP patients to demonstrate the involvement of these molecular pathways in humans. However, our group showed the anti-inflammatory potential of ES, with a significant reduction in proinflammatory cytokines (IL-6, IL-8) and proinflammatory lipid mediators [38].

The effectiveness of ES in preserving retinal function in RP patients has been shown by clinical parameters. A prospective, randomized, sham-controlled study evaluated the efficacy of weekly transcorneal ES (TES) for 6 consecutive weeks in RP patients. High electrical stimulation power induced a significant improvement of the VF area and electroretinogram (ERG) scotopic b-wave amplitude compared with the sham group [39]. In a group of patients followed for up to one year, an increase in the electric stimulation was associated with a trend of the prevention of VF and an improvement in the b-wave amplitudes of photopic ERG, also suggesting an impact on the cone photoreceptor system [40].

The beneficial effect of TES was further confirmed in a study including 101 RP patients treated for 30 min every week for 8 consecutive weeks. TES showed an increase in the mean BCVA and a significant VF improvement already one month after treatment when compared with baseline values, with good efficacy in peripheral retina [41]. To date, this study includes the highest number of patients treated with TES. Multifocal ERG recordings showed an increase in the amplitude and a shortening in the latency of p1 waves in the central rings, likely due to preserved cone function [41]. The visual function improvement after ES seems to be transient and partially disappeared at the 6 month follow-up after 4 months without treatment [41]. However, three RP patients who received three to six treatment courses of TES over 2.4 to 3 years documented significant improvements following each retreatment [42]. The frequency of treatment could be responsible for this different outcome. The determination of an ideal treatment frequency to ensure sustained visual improvement over time would optimize patient care.

The evaluation of specific biomarkers would be useful in clinical practice to understand the effectiveness of ES in order to modulate the treatment according to patient’s response. As photoreceptor death in RP results in a significant reduction in retinal metabolic function and oxygen consumption [43], the metabolic analysis and evaluation of oxygen saturation in retinal vessels would provide information on retinal cell survival [44]. Unfortunately, limited data are available on metabolic alterations induced in human eyes, likely due to the difficulty of non-invasive measurements. However, since the blood flow in the macular capillaries is influenced by the metabolic needs of the retina [45], several studies analyzed changes in the retinal blood flow (RBF) and in retinal oximetry (RO) following ES treatment. The latter are considered surrogate biomarkers of the biochemical changes found in preclinical studies. Bittner et al. found a significant improvement in the RBF in macular capillaries after 6 weeks of ES treatment [46]. The analysis of oxygen saturation in retinal arterioles and venules, along with the measurement of the arterio-venular difference in RP patients, can give an indication of oxygen consumption [44]. Two clinical studies estimated the effects of TES performed for 30 min once a week at 200% of the electrical phosphene threshold on RO. Six months after TES treatment, the mean retinal arteriolar oxygen saturation (A-SO_2_) increased from baseline, the venular saturation (V-SO_2_) decreased, and their difference (A-VSO_2_), which represents the oxygen consumption of the retina, showed a significant increase from baseline [47]. Even after 3 years of treatment, the A-VSO_2_ showed a slight incremental increase from baseline, albeit without reaching statistical significance [48]. Increased oxygen consumption indicates a metabolic response of the retinal tissue to ES. The delayed effect on the RBF suggests that this change is also mediated by molecular pathways rather than neural mediation.

ES appears to be a minimally invasive therapeutic strategy for RP patients to preserve as many retinal structures as possible, confirmed by its excellent safety profile. No serious side effects were reported, and all were resolved without sequelae [49,50], with dry eye sensation, likely due to direct contact between the electrode and the cornea, being the most common adverse event [50].

A literature search on ES suggests that it may have dual benefits: slowing disease progression in patients with early or intermediate stages of RP and stimulating visual function, particularly in those with advanced disease. However, randomized clinical studies are still needed to assess the efficacy of ES, both as a standalone treatment and in combination with other therapeutic strategies, the subset of patients that are most likely benefitting more, and in which stage of the disease this is happening, as well as to establish standardized treatment protocols.

### 3.2. Acupuncture

Acupuncture is part of complementary and alternative medicine. Over the years, this ancient Chinese practice has been increasingly considered as a treatment for pain, neuropathy, migraine, insomnia, and several other conditions [51]. Acupuncture has also been applied to ophthalmology, and several studies involving RP patients have provided evidence for its potential use in improving various aspect of visual function. A survey conducted in 2006–2007 on the use of complementary therapy in 96 RP patients found that 42% of respondents had tried acupuncture, of whom 61% indicated a subjective improvement in vision [52]. Studies conducted in the 1980s presented conflictual results in terms of the improvement of different parameters [53,54]. More recently, a prospective study of 12 adult RP patients evaluated the effects of a protocol combining electroacupuncture on the forehead and under the eyes and body acupuncture, showing improvements in scotopic sensitivity, an aspect of visual function not previously tested in studies of acupuncture and RP. Three subjects tested with the full-field stimulus testing (FST) had a better scotopic sensitivity in both eyes at one week after acupuncture, which was maintained for 10 to 12 months; two patients tested with scotopic sensitivity tester-1 showed a more rapid dark-adaptation; and four subjects reported subjective improvements in vision at night or in dark environments. However, no significant improvements in VA and VF were demonstrated [55]. From another prospective, interventional study patients received ten sessions of body acupuncture and reported significant improvements in UCVA and BCVA [56].

The potential beneficial effect of acupuncture may be related to molecular changes that occur in the retinal tissue in response to this practice. Studies on animal models of RP have linked electro-acupuncture to an increase in NGF, BDNF, and their receptor expression in the retinal tissue, increased outer retinal thickness, and enhanced vascularization [57]. Moreover, the positive effects of acupuncture have been further supported by visual cortical activation [58]. In humans, Bittner et al. showed significant improvement in retrobulbar central retinal artery mean flow velocity after 2 weeks of electro-acupuncture and increases in RBF after one month of treatment, compared to controls [46].

Despite the potential benefits, the evidence supporting the role of acupuncture in RP is still limited, as the existing studies often suffer from methodological flaws –small sample sizes, lack of control groups, and inconsistent treatment protocols. Considering the high safety profile, the low cost, and the relatively easy application, further research is desirable to clarify the potential role of acupuncture in managing RP and its use in clinical setting.

### 3.3. Oxygen and Ozone Therapy

Alternative therapeutic approaches to delay the progression of RP are available, although the rationale for their utilization is not completely established. Given the high oxygen demand of retina photoreceptors [43] and the correlation of reduced macular blood flow with diminished macular visual sensitivity in RP [59], hyperbaric oxygen may implement photoreceptor metabolic requests and lower damage progression. Daily hyperbaric oxygen therapy for up to two years resulted in a significant increase in ERG b-wave amplitudes [60], with maintained VA and VF compared to vitamin A treated patients, in the long term [61]. However, hyperbaric oxygen exposure can also cause a significant accumulation of reactive oxygen species, which was already elevated in RP with consequences on cell death and retinal degeneration [62].

Ozone therapy, potentially modulating oxidative stress by decreasing lipid peroxidation, represents an alternative approach [63]. Evidence on the use of ozone therapy is limited in clinical practice, although some studies suggest an improvement in VF and VA even in the long term, especially in early-stage disease patients [64].

Therefore, despite the promising results reported above, further studies are required to fully understand the benefits and potential side effects of these therapies.

## 4. Pharmacological Therapies

In the last 10–12 years, many pharmacological therapies have been used to improve retinal sensitivity or slow the progression of IRDs. The prostaglandin F2 α-agonist isopropyl unoprostone (IU), the calcium channel blocker nilvadipine, valproic acid, N-acetylcysteine, and growth factors have been tested in RP patients.

IU, a K channel activator, is used topically to treat glaucoma and has been reported to have neuroprotective effects on retinal neurons [65]. IU has been shown to significantly increase the blood flow in the optic nerve head, retinal microcirculation, and choroidal circulation in animals and humans [66], posing a rationale for its use in RP patients. Six months of topical treatment with IU twice a day showed an improvement in BCVA, central retinal sensitivity, and VF [67]. Topical IU was tested over one year, showing the preservation of macular sensitivity compared to controls [68]. Moreover, macular sensitivity improved in the IU treated eye when compared to the fellow untread eye. The up-regulation of ocular blood flow in the macular area and the direct neuroprotective effect were the mechanisms underlying the improvement [68].

Nilvadipine was shown to slow the progression of the central VF defect in RP in the long-term, irrespective of the calcium channel-blocking action [69].

Oral valproic acid (VPA), a broad-spectrum anticonvulsant, approved by the FDA for the treatment of bipolar disease and for migraine prophylaxis [70], inhibits the inflammatory response pathway through the apoptosis of microglial cells [71]. Recently, VPA has been suggested to reverse photoreceptor damage and to stimulate glial cells to differentiate into photoreceptor-like cells [72], downregulating complement proteins while upregulating various neurotropic factors. These properties make VPA a molecule with a unique biological profile suitable for treating retinal diseases, including retinal dystrophies [73,74]. An improvement in VA was reported upon one year of the VPA treatment of RP patients compared to controls [75]. A significant increase in the mfERG amplitude and a decrease in the latency in all fields was reported, correlated to the visual evoked response amplitude and latency, demonstrating the protective effects of VPA on photoreceptors and the therapeutic efficacy of the drug [75]. On the other hand, a prospective, placebo-controlled, double-masked study in patients with autosomal dominant RP failed to show clinical benefits of oral VPA, which may have a genotype-specific effect [76].

Nerve growth factor (NGF) is a crucial protein involved in the growth, maintenance, and survival of nerve cells, including those in the visual system. It has significant neuroprotective and neuroregenerative properties, in light of its ability to inhibit the apoptotic cascade [77], the final event leading to photoreceptor death, representing a promising therapeutic strategy in RP management. Falsini et al. tested the efficacy of NGF eye drops, administered for 10 days daily for a total dose of 1 mg NGF/pt, in patients with advanced RP [78]. Retinal function was assessed at baseline and after treatment by BCVA, macular focal electroretinogram (fERG) recording, and Goldmann visual field testing. Three patients reported a subjective feeling of improved visual performance; this was associated with a temporary enlargement of the VF in all three patients and to improved fERG in two of the three. A minority of patients experienced an improvement of visual performance [78]. Neither adverse events nor visual function losses were reported. Further studies are needed to confirm the potential effectiveness of NGF eye drops in RP patients.

More recently, in light of their anti-inflammation and immunoregulation functions, an intravenous infusion of human umbilical cord mesenchymal stem cells was tested in patients with advanced RP. Most patients improved their BCVA in the first 3 months after the infusion, and most of the patients (81.3%) maintained or improved their visual acuities for 12 months. The average VF sensitivity and flash visual evoked potential showed no significant difference. The treatment was safe with no serious local or systemic adverse effects in the whole follow-up and may be considered a promising therapeutic approach for advanced RP patients [79].

Rod photoreceptors degeneration is the first step in the onset of RP, followed by cone compromission by, among others, oxidative stress. N-acetylcysteine (NAC) has the ability to directly scavenge free radicals, thus reducing oxidative damage, and so can increase cone function/survival in RP. Different doses of NAC (600 mg to 1800 mg) administered twice daily in RP patients for 12 weeks and then three times a day for further 12 weeks resulted in a significant improvement in mean BCVA. A significant improvement in mean sensitivity over time was also reported in the cohort with the highest dose of NAC, but no significant change in mean EZ width was seen in any of the cohorts. NAC was safe and well tolerated and may improve sub-optimally functioning macular cones, although further studies are required to determine its long-term stabilization and/or improvement in visual function in patients with RP [80].

Overall, various pharmacological approaches have been investigated in recent years, most of which have focused on anti-inflammatory effects. These compounds hold potential for slowing disease progression and preserving visual acuity for as long as possible. However, further clinical studies are needed to fully explore their effectiveness and optimize their use in treating RP.

## 5. Lifestyle and Nutrients

While the genetic basis of RP is well understood, the influence of lifestyle factors and the therapeutic potential of specific nutrients on disease progression remains a subject requiring deeper investigation.

### 5.1. Physical Activity and Smoking

Physical activity has long been recognized for its beneficial effects on overall health and well-being. Preclinical research showed that in a mouse model of RP, exercise had a positive effect on retinal degeneration, preventing vision loss and retinal damage, with an increased number of cone cells [81], decreased loss of photoreceptors, and reduced retinal inflammation [82]. Physical activity in RP patients increased self-reported visual function and QoL in a preliminary study [83].

Smoking is a well-established risk factor for various systemic and ocular diseases and has emerged as a potential contributor to the exacerbation of retinal degeneration in RP. Indeed, a retrospective study showed that VA was notably worse in smoking patients compared to non-smokers. Smokers also exhibited a thinner ellipsoid zone and central retinal compared to non-smokers, although with no statistical significance [84].

### 5.2. Fatty Acids

Some natural compounds have also been evaluated to treat RP, on the basis of their involvement in different enzyme/molecular pathways that are altered or defective in the onset of the disease.

Fatty acids, as a part of the membrane of photoreceptors, are central components of retinal visual cycle and phototransduction functioning. In patients with RP, the polyunsaturated fatty acid docosahexaenoic acid (DHA) is decreased in both mouse models and human patients. DHA is known to play a critical role in the development and maintenance of the retina. However, the relationship between reduced DHA levels and retinal degeneration is complex and not fully understood, due also to the complex and different lipid composition of rods and cones [85].

### 5.3. Antioxidants

Oxidative stress, resulting from an imbalance between the production of reactive oxygen species and the body’s capacity to neutralize them with antioxidants, has been linked to various neurodegenerative diseases, including RP. The retina of RP patients is prone to oxidative damage, as genetic changes may cause the death of rod cells, which are highly susceptible to oxidative stress, secondary degeneration of the remaining cone cells, and progressive retinal degeneration [62].

A therapy with antioxidants has been suggested for retinal dystrophies, and preclinical studies confirmed that a combination of antioxidants effectively reduced oxidative damage in the retina of a mouse model for RP, correlating with a significant increase in the number of surviving cones [86,87].

Clinical studies with antioxidants are still preliminary. For instance, chlorogenic acid (CGA), a polyphenol found in agricultural products, with antibacterial, anti-inflammatory, antioxidant, and anti-carcinogenic activities [88], has been tested in RP patients. CGA supplementation resulted in a significant increase in multifocal electroretinography, supporting the possibility of a beneficial effect on the peripheral area at the margins of the retinal degeneration [89]. However, CGA failed to show a significant improvement in the visual outcome [89]. Similarly, *Lycium barbarum* L. supplementation showed some potential protection from the thinning of the macular layer, but functional testing failed to show differences from the controls [90]. Other polyphenols, such as the flavonoid quercetin, are gaining interest, given the potential role in maintaining the proper folding of rhodopsin; however, clinical research in this area is still limited [91].

Vitamin A is a group of unsaturated nutritional compounds that includes retinol, retinal, and retinoic acid and provitamin A carotenoids. Among these, β-carotene is considered the most important provitamin A carotenoid. In the retina, vitamin A is an essential chromophore, regenerating visual pigments after photoactivation, a crucial process for maintaining normal vision. Adequate vitamin A intake is therefore required for retinal health and function. While some studies report a protective role in the loss of cone function for vitamin A [92], others have not shown any specific effect upon its administration [93]. A retrospective analysis focused on children with RP revealed that the vitamin A cohort exhibited a significant slower exponential rate of decline in cone ERG amplitude compared to the control cohort. The relatively small sample size does not allow any definitive conclusions regarding the long-term efficacy of vitamin A supplementation [94]. Actually, a metanalysis on the results from the studies about vitamin A and DHA concluded that there is currently no clear evidence for the benefit of treatment with such treatments for RP patients, as large-scale, randomized controlled trials are missing [95].

The 9-cis β-carotene is a precursor of the retinoid 9-cis-retinal, which present a similar light absorption spectrum to the one of 11-cis-retinal, a central molecule in the healthy retinoid cycle of the retinal pigment epithelium. The administration of 9-cis β-carotene allowed the incremental increase in retinal function, paving the road for its potential use for some RP patients [96].

### 5.4. Lutein

Lutein, a xanthophyll and the primary carotenoid accumulated in the human macula to form macular pigment, possesses antioxidant properties that could be beneficial for retinal health. However, its specific role in retinitis pigmentosa is not fully understood.

Studies on animal models have explored its protective effects RP, with promising results in preventing photoreceptor degeneration. Treatment with lutein and zeaxanthin, another xanthophyll, exhibited larger a-wave and b-wave amplitudes in dark-adapted ERG, as well as larger b-wave amplitudes in light-adapted ERG [97]. Moreover, lutein administration was associated with a significant increase in outer nuclear layer thickness in mice [98]. Different results come from studies in humans, where daily lutein supplementation did not lead to significant improvements in VA or VF sensitivity, with only modest ERG improvement and a slowed loss of the midperipheral visual field [99].

### 5.5. Curcumin

Curcumin, the bioactive compound derived from the plant turmeric, or *Curcuma longa*, has gained interest due to its potential multifaceted pharmacological effects, including anti-inflammatory, antioxidant, and neuroprotective properties. Curcumin suppresses the production and release of proinflammatory cytokines, chemokines, and enzymes implicated in retinal inflammation, and preclinical studies reported the preservation of retinal structure, the increased thickness of the outer and inner nuclear layers, and improved ERG responses [100].

The research surrounding lifestyle factors and nutritional interventions, despite holding potential for RP treatment, still presents challenges in translating pre-clinical findings into robust clinical outcomes. The complex nature of RP and the heterogeneity of patient responses are factors to be considered when planning clinical approaches to test these interventions. Future research should elucidate the specific mechanisms by which oxidative stress leads to cone cell death and retinal degeneration, and longitudinal studies incorporating advanced imaging techniques and functional assessments will provide critical insights into the dynamics of retinal changes and treatment outcomes over time.

## 6. Psychological Approach

In patients with retinal dystrophies, the interplay between psychological factors such as anxiety, fear, and a decline in QoL can exacerbate the condition, leading to a negative loop where psychological distress may influence the disease’s course. Fear, stress, and depression can arise from the awareness of one’s decreasing ability to engage in daily activities and social interactions [101]. In addition, RP has been linked with the onset of mental health problems and with the fear of stigma due to psychiatric issues, triggering a negative psycho-physical feedback and thus contributing to a sense of loss of identity and social isolation [102]. Patients with a more fragile mental health may need further support to reduce the impact of mental illnesses derived from RP. RP and progressive visual disability can also have a significant economic impact on patients influencing healthcare, personal support costs, a loss of working hours, working quality, and income [103,104,105].

In addition, comorbidities such as hyperglycemia, ocular hypertension, hyperlipidemia, and obesity can be favored by increased stress and depression and may lead to increased systemic oxidative stress that may worsen the progression of retinal diseases [106].

Some studies quantified the degree of visual impairment impacting the decreased QoL, pointing out that exceeding some thresholds of VF and VA (<20° and <0.3, respectively) correlated with a significant decrease in the ability to carry out daily activities, which, in turn, worsens the QoL [107].

Effective stress management and psychological interventions can potentially improve QoL and may influence the disease’s trajectory. By addressing the emotional and psychological needs of RP patients, healthcare providers can help mitigate the adverse effects of anxiety and fear, ultimately supporting better health outcomes. Multidisciplinary actions are therefore suggested to initiate rehabilitation and support the patients to translate the functional benefit obtained with therapy in the improvement of the QoL. A preventive and personalized intervention acting on the negative influence caused by the progressive loss of sight is suggested to support patients’ QoL and functional independence.

## 7. Discussion

**Figure 1:**
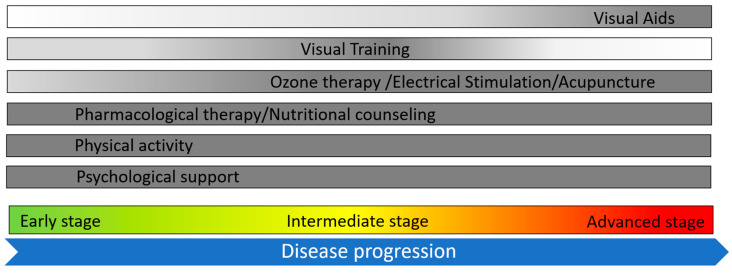
Proposed multidisciplinary management framework for retinitis pigmentosa (RP). The integration of various therapeutic and supportive interventions at different phases of disease progression are suggested for managing RP and enhancing patients’ QoL. The color intensity of the gray bars is proportional to the degree to which the corresponding intervention is applied along the course of disease progression.

The treatment landscape for RP is evolving, with gene therapy emerging as a promising approach. Currently, the only approved gene therapy is voretigene neparvovec, which targets mutations in the *RPE65* gene [9]. As this therapy addresses only one of the several genetic mutations that cause RP, there is a significant gap in the availability of treatments for the broader patient population. A range of potential interventions that may help manage the progression of the disease are reported in the literature, and, although not curative, they are currently being applied and explored in the clinical practice. These include pharmacological treatments, aiming to slow retinal degeneration, as well as non-pharmacological interventions such as visual rehabilitation, electrostimulation, ozone therapy, and acupuncture. Additionally, photo-selective filters have been explored for their potential to enhance residual vision and protect retinal cells from further damage. It is difficult to draw definitive conclusions on the efficacy of these approaches, since, as already highlighted above, at the closing comments of almost every thematic area, further investigation and more in-depth studies are due. On top of that, the matter of a possible different effect on the diverse genetic variants should be considered more by the studies since the point seems underestimated; for instance, through the background of patients recruited, only few studies took into account the genotypes [24,49,55,76,78,83], but only in one study [76] was used as inclusion criteria to evaluate the effect of VPA in a homogeneous cohort. However, given the ease of application and the absence of adverse events for many of these interventions, and despite the varied levels of effectiveness, they should be considered and potentially included in the clinical management of RP to offer patients tools to improve their QoL. Ophthalmogenetic clinics should therefore be organized not only for the correct diagnosis of the IRD, but to offer patients a holistic approach. IRD experts and geneticists should work side-by-side with an ambulatory to offer low-vision services; this could include the presence of an orthoptist suggesting visual rehabilitation or the use of visual aids, according to the patient’s characteristics and needs and to the stage of the disease (Figure 1), providing meaningful support to those living with progressive vision loss. Additionally, therapies like electrostimulation, which have shown promise in some studies, should be considered as part of a comprehensive care plan. Nutritional counseling, guided by a dietitian, could also play a role in optimizing a patient’s lifestyle and possibly influencing disease progression. Psychological support, given the emotional burden associated with vision loss, is another vital component of patient care, with a particular focus on stress anxiety, depression, and their negative impact on the degeneration. The insights gathered from the literature strongly suggest the multidisciplinary management of RP, with a holistic approach involving ophthalmologists, orthoptists, rehabilitation experts, dietitians, and psychologists to address the various needs of RP patients. This framework will ensure that, while waiting for more widespread gene therapy solutions, patients receive comprehensive care aimed at preserving their QoL and potentially slowing the disease’s progression.

## Data Availability

No new data were created or analyzed in this study. Data sharing is not applicable to this article.
